# Lichtenstein inguinal hernia repairs with porcine small intestine submucosa: a 5- year follow-up. a prospective randomized controlled study

**DOI:** 10.1093/rb/rbaa055

**Published:** 2021-01-04

**Authors:** Baoshan Li, Xin Zhang, Yi Man, Jiadong Xie, Wei Hu, Huang Huang, Yinlong Wang, Hongguang Ma

**Affiliations:** Department of Hernia and Abdominal Wall Surgery, Tianjin Union Medical Center, 190, jieyuan Road, HongqiaoDistrict, Tianjin 300121, China; Department of Hernia and Abdominal Wall Surgery, Tianjin Union Medical Center, 190, jieyuan Road, HongqiaoDistrict, Tianjin 300121, China; Department of Hernia and Abdominal Wall Surgery, Tianjin Union Medical Center, 190, jieyuan Road, HongqiaoDistrict, Tianjin 300121, China; Department of Hernia and Abdominal Wall Surgery, Tianjin Union Medical Center, 190, jieyuan Road, HongqiaoDistrict, Tianjin 300121, China; Department of Hernia and Abdominal Wall Surgery, Tianjin Union Medical Center, 190, jieyuan Road, HongqiaoDistrict, Tianjin 300121, China; Department of Hernia and Abdominal Wall Surgery, Tianjin Union Medical Center, 190, jieyuan Road, HongqiaoDistrict, Tianjin 300121, China; Department of Hernia and Abdominal Wall Surgery, Tianjin Union Medical Center, 190, jieyuan Road, HongqiaoDistrict, Tianjin 300121, China; Department of General Surgery, China-Japan Friendship Hospital, Sakura Garden East Street, Chaoyang District, Beijing 100029, China

**Keywords:** inguinal hernia, Lichtenstein, SIS Biological patch, recurrence, infection, chronic pain

## Abstract

Porcine small intestine submucosa (SIS) biologic patch has been used in inguinal hernia repair. However, there are little data available to assess the long-term effect after repair. This study aimed to explore the long-term effect of SIS patch in open inguinal hernia repair. Sevent-six patients with unilateral inguinal hernia were treated with Lichtenstein tension-free hernia repair using SIS patch (Beijing Datsing Bio-Tech Co., Ltd.) and Surgisis patch (COOK, USA) in Tianjin Union Medical Center and China-Japan Friendship Hospital. In the trial, the long-term efficacy of the treatment group and the control group were compared. A total of 66 patients in both groups received long-term follow-up (> 5 years) after surgery, with a follow-up rate of 86.8%. During the follow-up period, there was one case of recurrence, one case of chronic pain in the control group. There was no statistically significant difference (*P* > 0.05) in terms of recurrence, chronic pain, foreign body sensation and infection between the two groups of patients. After long-term observations, it has been found that the porcine small intestinal submucosa (SIS) biological patch is safe and effective for inguinal hernia Lichtenstein repair, and has a low recurrence rate and complication rate.

## Introduction

Inguinal hernia is a common and frequently-occurring disease in surgery, and its etiology is multifactorial. Among them, connective tissue changes play an important role in the pathogenesis of inguinal hernia [1], so artificial patch has been widely used in the field of hernia surgery. The use of artificial patch has greatly reduced the recurrence rate after inguinal hernia surgery. However, its related complications, such as acute, chronic infection, chronic pain, foreign body sensation in the inguinal area, erosion of adjacent organs and so on, have attracted more and more attention [2, 3]. Biological patch has become a new research hotspot in the field of hernia surgery due to its good histocompatibility, rapid vascularization, resistance to infection, and degradability. There have been many reports on the safety and short-term effectiveness of biologic patch [4–7]. However, reports of follow-up data from long-term, prospective, randomized controlled trials over three years have remained scarce.

Tianjin Union Medical Center and China-Japan Friendship Hospital conducted double-center, single-blind, prospective, randomized controlled trials between August 2013 and March 2014. In the trial, 76 patients with unilateral inguinal hernia were treated with Lichtenstein tension-free hernia repair using small intestinal submucosa (SIS) patch from Beijing Datsing Bio-Tech Co., Ltd. and Surgisis patch from COOK, USA. The trial used non-inferior design to evaluate the clinical effect and safety of domestic SIS patch. The preliminary results were published in 2017, and the short-term effects and safety were satisfactory [8]. At present, the trial has been completed for more than 5 years. We followed up the above cases again to evaluate the long-term effect of SIS patch in the open tension-free repair of inguinal hernia.

## Materials and methods

### General materials

The sample size of this study was 76 cases, while 38 cases were respectively selected in the control group and treatment group according to the random table. There were 35 males and 3 females in the control group, aged (56 ± 15) years old, and BMI (23 ± 2) kg/m^2^; 34 males and 4 females in the treatment group, aged (54 ± 13 years old), and BMI (22.86 ± 2.15) kg/m^2^. There was no statistically significant difference (*P* > 0.05) in the basic conditions of patients in the two groups.

### Inclusion criteria

Adults, Gilberts classification, patients with type I–VI Primary unilateral inguinal hernia, non-emergency surgery.

### Exclusion criteria

Patients with recurrent hernia and incarcerated hernia; patients with systemic or local infection; patients with ascites due to cirrhosis of the liver; patients undergoing radiation and chemotherapy; patients taking immunosuppressive therapy; patients who are pregnant or breastfeeding; patients with religious taboos or allergic to porcine body materials.

### Information on the use of patch

The control group used the Biodesign Surgisis patch from COOK, USA, the product model was Ib63766602, c-ihm-8 × 15p; the treatment group used SIS patch produced by Beijing Datsing Bio-Tech Co. Ltd., product model was 13022601-0009, 8 × 15 cm.

The clinical trial protocol and related data for clinical trials were implemented after being reviewed and approved by the ethics committee of the clinical trial institution. The subjects fully understood the trial content and signed a written informed consent form.

### Random method

Before the test, the patch number was assigned according to the random table, and the patients were randomly assigned to the treatment group and the control group according to the time sequence of the patients’ enrollment and the patch number. The patients were single-blind and the followers were not surgeons.

### Surgical technique 

All surgeries were performed by a skilled surgeon (independently completed Lichtenstein surgery> 1000 cases). A local anesthesia or spinal anesthesia was adopted according to the opinions of patients and anesthesiologists. An inguinal incision was taken and the external oblique aponeurosis was opened. The spermatic cord was freed by blunt and sharp dissection from the underlying posterior inguinal canal. A search for an indirect hernia sac was started, dissected completely or transected the hernia sac. The indirect hernia sac was ligated at the level of internal ring, narrow internal ring by absorbable suture. When a direct hernia was found, the hernia sac was reduced with a purse string of absorbable suture at the base of the hernia sac. One side of the inguinal ligament was sutured continuously from the pubic tubercle with prolene thread under the inner ring after laying biologic patch. The upper part is fixed by intermittent suture with prolene thread to avoid damaging the iliac hypogastric nerve and iliac inguinal nerve. If the nerve distribution affects the placement and fixation of the patch, the part of nerve needs to be cut off. The aponeurosis, subcutaneous and skin by layer were sutured with absorbable suture. The control group used the Surgisis patch from COOK, USA; the treatment group used the SIS patch produced by Beijing Datsing Bio-Tech Co. Ltd.

### Follow-up and observation indices

All patients came to the hospital for reexamination and follow-up in a short period (within 24 weeks) after surgery; long-term follow-up was conducted by phone, SMS or mail. The death or recurrence of the patient due to other reasons is regarded as the end point of this follow-up. And the follow-up lasted until 26 December 2019. Observation indices include recurrence, chronic pain, foreign body sensation and infection. Postoperative chronic pain and foreign body sensation were assessed using a simple verbal scale (SVS). The SVS score was 4 levels: none, mild, moderate and severe [6].

### Statistical methods

This trial used SPSS19.0 software for data statistics. The comparison of count data was performed by χ^2^ test and expressed by rate; the comparison of measurement data was performed by t test and expressed by *x*^–^ ± s. *P* < 0.05 was considered as statistically significant difference.

## Results

### Comparing clinical data and short-term efficacy between two groups of patients

There was no statistical significance between the two groups in terms of anesthesia method, surgical site, Gilbert classification and surgical time (*P* > 0.05). See Table 1. The cure rate was 100% in the control group and the treatment group. No swelling was found in the groin operation area at rest and coughing state during the 1, 4, 24 weeks of follow-up, and no recurrence occurred in the two groups at 24 weeks (6 months). In the control group, five patients had abnormally increased white blood cells and localized swelling and redness in the wound. Of these, four patients had mild fever and recovered shortly without special treatment. No serious adverse events occurred in both groups; the incidence of adverse reactions in the treatment group was 0. There was no statistically significant difference between the two groups (*P* = 0.054). See Table 2.

**Table 1.** Comparison of intraoperative conditions between two groups of patients

Group	Case	Anesthesia method (case)		Surgical site (case)		Gilbert type (case)					Operation time (min, *x*^–^ ±S)
		Spinal anesthesia	Local anesthesia	Left	Right	I	II	III	IV	V	
Control group	38	22	16	16	22	9	16	0	11	2	49 ± 15
Treatment group	38	17	21	14	24	9	21	1	7	0	53 ± 21
*P* value		0.359		0.815		0.335					0.347

**Table 2.** Comparison of general data and short-term postoperative efficacy between two groups of patients

Group	Case	Age (year)			Short-term complications after surgery				
		Average	<60	≥60	Local swelling	Fever	Recurrence	Incision infection	Chronic pain
Control group	38	56.3	20	18	5	4	0	0	0
Treatment group	38	53.9	22	16	0	0	0	0	0
	76	55.1	42	34	5	4	0	0	0

### Long-term observations on efficacy

A total of 66 patients received long-term follow-up, the follow-up rate was 86.8%, and the average follow-up time was 68.7 (65–75) months. Among the patients who received long-term follow-up, one patient in the control group developed a recurrent hernia after 7 months of post-surgery follow-up (the second operation confirmed a new direct hernia, which was repaired by artificial patch repair). One patient had persistent pain due to strenuous exercise 2 years after the operation (the symptoms gradually disappeared after 1 year of drug treatment), no foreign body sensation case, and no infection case. The treatment group had no cases of recurrence, chronic pain, foreign body sensation and infection cases. There was no statistically significant difference (*P* > 0.05) in terms of recurrence, chronic pain, foreign body sensation and infection between the two groups, as shown in Table 3.

**Table 3.** Comparison of long-term postoperative efficacy between two groups of patients

	Case	Follow-up (deceased)	Follow-up time (months)	Recurrence	Chronic pain	Foreign body sensation	Postoperative infection
Control group	38	35 (4)	68.77 ± 2.65	1	1	0	0
Treatment group	38	31 (0)	68.75 ± 2.65	0	0	0	0
*P* value				0.42	0.42		
	76	66 (4)	68.77 ± 2.65	1	1	0	0

## Discussion

Inguinal hernia is one of the most common diseases in surgery. In the development of hernia repair surgery, the popularity of tension-free hernia repair is a milestone in the development of inguinal hernia surgery. Lichtenstein surgery is a classic application for tension-free hernia repair. Artificial patch made of polypropylene plays a vital role in tension-free inguinal hernia repair, which has been used for nearly 30 years. Artificial patch repairs hernia defects by inducing foreign body reaction and mechanical barrier effect, which greatly reduces the recurrence rate of inguinal hernia. However, the long-term adverse effects related to patch also weaken its advantage. As a result, surgeons continue to explore new materials.

The rise of biological patch in recent years is a hot topic in the field of hernia and abdominal wall surgery. Biological patches are mainly derived from the skin, pericardium, intestine, dura mater and other tissues of animals (xenogeneic) and humans (allogeneic). After being processed and preserved by various methods, all kinds of cells and antigens contained in the tissue are removed, and the collagen-rich of 3D fiber framework is retained, which can be used for host cell proliferation, tissue remodeling and vascular regeneration, supporting and strengthening tissue repair [4, 9]. Some studies have shown that SIS bio-patch acts as a biological barrier in the body. As fibroblasts migrate and proliferate, it acts as a scaffold for the growth of new tissues. And its degradation is very fast, and it is gradually remolded by the host, resulting in a completely host derived repair tissue structure. SIS biological patch has the advantages of strong resistance to infection, good biocompatibility, no excessive scar tissue formation and no long-term chronic pain. Its safety and short-term effectiveness in inguinal hernia repair are widely recognized [10–13].

### The significance of long-term follow-up with biological patch

Although the short-term effects of biologic patch are positive, many clinicians are concerned about the long-term effects of biologic patch, especially the recurrence rate [14]. Because of the lack of long-term follow-up reports of biological patch, especially the lack of long-term follow-up data from multicenter, large-sample randomized controlled studies, clinicians are often very cautious in the use of biological patch. Instead, the biological patch is only used in some special circumstances, such as young people not having children, potential infection, upon request by patients and clinical trials. This study started in August 2013 and ended in December 2019. The shortest follow-up period has been more than 5 years. Sixty-six of the 76 cases have obtained long-term follow-up, with a follow-up rate of 86.8%. The follow-up times and results give us great confidence in the validity of our claims.

### There was no statistical difference between the long-term results of two kinds of biological patches

The original purpose of this trial was to select the nationally popular Surgisis patch produced by COOK, USA as a control group, using a non-inferior design, to study the clinical safety and effectiveness of SIS biological patch produced by Beijing Datsing Bio-tech Co. Ltd. Among the patients who received long-term follow-up, the control group had one case of relapse 7 months after surgery, one case of long-term pain, no infection case and foreign body sensation case; while there was no recurrence, chronic pain, infection, or foreign body sensation in the treatment group, and there was no statistical difference in terms of long-term complications between the two groups. In the control group, a second operation was performed after the recurrence. The primary diagnosis was indirect inguinal hernia, and the secondary operation confirmed that it was a new direct hernia. One case of chronic pain occurred in the control group 2 years after the operation, which was persistent pain due to strenuous exercise, and the symptoms gradually disappeared after 1 year of drug treatment. In summary, it can be shown that the long-term effect of clinical application of SIS patch from Datsing Bio-tech and Surgisis patch from COOK Company is similar, and there is no statistical difference.

### The long-term results of small intestinal submucosal biologic patch are satisfactory

The SIS patch is taken from the porcine small intestine submucosa. After physical, chemical and enzymatic treatment, the extracellular matrix complex collagen and some growth factors formed by cellular components and antigens are removed. As a biological scaffold for host tissue and cell reconstruction, it has the advantages of complete absorption and good histocompatibility [15]. The two patches in this test are derived from the submucosa of the porcine small intestine, which have the same tissue source, similar structure and function, and there is no statistical difference in the test subjects. There is also no statistical difference in short-term and long-term efficacy in this test. The total recurrence rate of the two groups combined as a small intestinal submucosal biologic patch was 1.52%. Ravo *et al.* [16] followed up 101 patients for 10 years after performing strengthening repair with SIS biological patch on the basis of repairing transverse abdominal fascia. The results showed that the recurrence rate of inguinal hernia was only 1.9%, and the results of the two studies were similar. Although the recurrence rate of biological patches is higher than that of artificial patches [17], such long-term recurrence rate results can be accepted by clinicians and patients. Chronic pain and foreign body sensation after inguinal hernia repair are two other common complications that affect life quality of patients. Among the patients followed up in this study, one patient had chronic pain in the surgical area (moderate SVS level) due to strenuous exercise 2 years after surgery. After oral medication, the symptoms gradually subsided. The occurrence is considered to be related to the injury of strenuous exercise, and the relationship with the biological patch is not clear. The rest of the patients recovered well after surgery without long-term chronic pain and foreign body sensation. So this result is significantly better than that of artificial patches [4]. In addition, with the increasing application time of artificial patches in inguinal hernias, more and more chronic infections appear, and infections can even occur 9 years after surgery. In most cases, it is necessary to remove the patch again [18]. One of the great advantages of biological patches is that with the migration and proliferation of fibroblasts, as a scaffold for the growth of new tissues, biological patches will be gradually degraded and absorbed without leaving foreign bodies, so the long-term chronic infection may be significantly lower than artificial patch. The 66 patients followed in our two groups did not develop infection for more than 5 years after surgery. The results of this long-term follow-up will boost clinician confidence in the biologic patch.

## Conclusion

In summary, the results of this test indicate that the application of SIS biologic patch from Beijing Datsing Bio-tech Co., Ltd. to inguinal hernia Lichtenstein repair is similar to Surgisis patch from COOK Company. After the porcine small intestinal submucosal biologic patch was applied to inguinal hernia Lichtenstein repair, its long-term effect was satisfactory through the long-term follow-up observation of its recurrence rate, chronic pain, and foreign body sensation and infection rate.

### Limitations

First, the sample size of the study is limited, and more large clinical samples are needed to confirm this result. Second, our research focuses on the application of biological patch in open inguinal hernia repair. Before placing the biological patch, we first narrowed the inner ring of the indirect hernia or reduced the direct hernial sac with eversion, which is different from laparoscopic biologic patch inguinal hernia repair. And studies have shown that the latter has a high recurrence rate [19, 20], which may be due to the existence of defects affecting cell proliferation and reconstruction. Further research is needed on the applicability and use of laparoscopic inguinal hernia repair (TAPP/TEP). 

*Conflict of interest statement.* None declared. 

## References

[1] Öberg S , Andresen K, Rosenberg J. Etiology of inguinal hernias: a comprehensive review. Front Surg 2017;4:52. 29018803 10.3389/fsurg.2017.00052 PMC5614933

[2] Kouhia S , Vironen J, Hakala T et al Open mesh repair for inguinal hernia is safer than laparoscopic repair or open non-mesh repair: a nationwide registry study of complications. World J Surg 2015;39:1878–86. 25762240 10.1007/s00268-015-3028-2

[3] Jianxiong T , Lei Z, Shaojie L. Current status of hernia and abdominal wall surgery and innovation research and development of repairing materials [J]. Chin J Dig Surg 2018;17:1071–5.

[4] Fang Z , Ren F, Zhou J et al Biologic mesh versus synthetic mesh in open inguinal hernia repair: system review and meta-analysis. ANZ J Surg 2015;85:910–6. 26183816 10.1111/ans.13234

[5] Arslani N , Gajzer B, Papeš D et al A new approach for transversalis fascia reinforcement in Lichtenstein's inguinal hernia repair. Surg Today 2013;43:211–4. 22718088 10.1007/s00595-012-0232-7

[6] Ansaloni L , Catena F, Coccolini F et al Inguinal hernia repair with porcine small intestine submucosa: 3-year follow-up results of a randomized controlled trial of Lichtenstein's repair with polypropylene mesh versus Surgisis Inguinal Hernia Matrix. Am J Surg 2009;198:303–12. 19285658 10.1016/j.amjsurg.2008.09.021

[7] Bochicchio GV , Jain A, McGonigal K et al Biologic vs synthetic inguinal hernia repair: 1-year results of a randomized double-blinded trial. J Am Coll Surg 2014;218:751–7. 24655865 10.1016/j.jamcollsurg.2014.01.043

[8] Jie Z , Xiaoliang S, Yinlong W et al Stage III clinical trial on the application of porcine small intestine submucosa mesh in inguinal hernia repair [J]. Chin J Hernia Abdominal Wall Surg (Electronic Edition) 2017;11:344–7.

[9] Yan G , Jianjun Y, Zhicheng S et al The importance of biological mesh repair in adolescent inguinal hernia repair [J]. Chin J Pract Surg 2019;39:803–6.

[10] Bellows CF , Shadduck P, Helton WS et al Early report of a randomized comparative clinical trial of Strattice™ reconstructive tissue matrix to lightweight synthetic mesh in the repair of inguinal hernias. Hernia 2014;18:221–30. 23543334 10.1007/s10029-013-1076-9

[11] Nie X , Xiao D, Wang W et al Comparison of porcine small intestinal submucosa versus polypropylene in open inguinal hernia repair: a systematic review and meta-analysis. PLoS One 2015;10:e0135073. 26252895 10.1371/journal.pone.0135073 PMC4529205

[12] Sun L , Chen J, Shen Y. Randomized controlled trial of Lichtenstein repair of indirect inguinal hernias with two biologic meshes from porcine small intestine submucosa. Ther Clin Risk Manag 2019;15:1277–82. 31802878 10.2147/TCRM.S208185 PMC6827516

[13] Shaojie L , Jianxiong T, Shaochun L et al Clinical analysis on the technique of transverse fascia reinforcement with biological mesh in young inguinal hernia patients [J]. Chin J Pract Surg 2019;39:821–4.

[14] Öberg S , Andresen K, Rosenberg J. Absorbable meshes in inguinal hernia surgery: a systematic review and meta-analysis. Surg Innov 2017;24:289–98. 28492358 10.1177/1553350617697849

[15] Ansaloni L , Catena F, Gagliardi S et al Hernia repair with porcine small-intestinal submucosa. Hernia 2007;11:321–6. 17443270 10.1007/s10029-007-0225-4

[16] Ravo B , Falasco G. Pure tissue inguinal hernia repair with the use of biological mesh: a 10-year follows up. A prospective study. Hernia 2020;24:121–6. 31111323 10.1007/s10029-019-01976-y

[17] Yinlong W , Xin Z, Jiadong X. Lichtenstein procedure for inguinal hernia: a single center experience of 5795 cases report[J]. J Surg Concepts Pract 2013;18:218–23.

[18] Fenglin Z , Changfu Q, Jie C et al Can the wounds after infected meshes removal be sutured primarily: a retrospective study [J]. Chin J Gen Surg 2017;32:332–5.

[19] Alshkaki G. Placement of a non-cross-linked porcine-derived acellular dermal matrix during preperitoneal laparoscopic inguinal hernia repair. Int Surg 2013;98:133–9. 23701148 10.9738/CC176 PMC3723178

[20] Ho CH , Liao PW, Yang SS et al The use of porcine small intestine submucosa implants might be associated with a high recurrence rate following laparoscopic herniorrhaphy. J Formos Med Assoc 2015;114:216–20. 23725634 10.1016/j.jfma.2013.03.007

